# History of COVID-19 Symptoms and Seroprevalence of SARS-CoV-2 Antibodies in HIV-Infected Patients in Northern France after the First Wave of the Pandemic

**DOI:** 10.3390/microorganisms9122491

**Published:** 2021-12-01

**Authors:** Agnès Meybeck, Thomas Huleux, Macha Tétart, Pauline Thill, Vincent Derdour, Laurence Bocket, Enagnon Kazali Alidjinou, Pierre Patoz, Olivier Robineau, Faiza Ajana

**Affiliations:** 1 Service des Maladies Infectieuses et du Voyageur, Centre Hospitalier de Tourcoing, 59200 Tourcoing, France; thuleux@ch-tourcoing.fr (T.H.); mtetar@ch-tourcoing.fr (M.T.); pthil@ch-tourcoing.fr (P.T.); vderdour@ch-tourcoing.fr (V.D.); ppatoz@ch-tourcoing.fr (P.P.); orobineau@ch-tourcoing.fr (O.R.); fajana@ch-tourcoing.fr (F.A.); 2 Laboratoire de Virologie, CHRU de Lille, 59000 Lille, France; laurence.bocket@chru-lille.fr (L.B.); enagnonkazali.alidjinou@chru-lille.fr (E.K.A.)

**Keywords:** COVID-19, SARS-CoV-2, prevalence, risk factors, HIV, epidemiology

## Abstract

To assess the prevalence of COVID-19 in people living with HIV (PLWHIV), we performed an epidemiological survey from 1 April through 1 August 2020 in an HIV reference center in Northern France. PLWHIV completed a questionnaire about risk exposures and symptoms consistent with COVID-19 and performed a SARS-CoV-2 serology. Among the 600 PLWHIV included, 16 have been infected with SARS-CoV-2. Symptoms consistent with COVID-19 were frequent both in SARS-CoV-2 positive and negative patients (67% vs. 32%, *p* = 0.02). Among SARS-CoV-2 infected patients, one (6%) has been hospitalized and five (31%) have been asymptomatic. Close contact with a confirmed COVID-19 case was the only factor associated with COVID-19 acquisition (40% vs. 13%, *p* = 0.01). The prevalence of COVID-19 in PLWHIV was 2.5%, half of the overall population estimate after the first wave of the pandemic in France. In conclusion, proportion of asymptomatic COVID-19 was high in PLWHIV. The prevalence of COVID-19 in PLWHIV was two times lower than in the general population.

## 1. Introduction

In December 2019, a novel coronavirus, severe acute respiratory syndrome coronavirus 2 (SARS-CoV-2), emerged in the city of Wuhan in China, and resulted in the coronavirus disease 2019 (COVID-19) pandemic [1]. The disease can be asymptomatic, induce mild symptoms including cough and fever, or lead to acute respiratory distress syndrome (ARDS), sepsis, multi-organ failure, and death [2,3]. A few previously published studies suggested that people living with HIV (PLWHIV) with well-controlled disease experienced comparable COVID-19 clinical presentation and outcomestothe general population [4,5,6]. Knowledge about epidemiology in PLWHIV islimited [7]. Immunodeficiency in PLWHIV may increase their susceptibility to COVID-19 as withother infectious diseases [8]. Moreover, stigma and poverty could favour COVID-19 transmission in PLWHIV. In France, more than half of the PLWHIV reported economical or social deprivation. On the other hand, people identified as at risk could be more cautiousconcerning social distancing measures. Several antiretroviral drugs have shown in vitro activity against SARS-CoV-2 and could have a protective role against COVID-19 in PLWHIV currently receiving these drugs [9,10]. In France, the extension of the epidemic led to stringent containment measures. Lockdown was maintained from 17 March to 11 May 2020. During this period, the COVID-19 testing national strategy was centred on symptomatic infections, especially hospitalized patients and symptomatic healthcare workers, while most paucisymptomatic and asymptomatic cases were not confirmed [11]. The incidence of COVID-19 may have been underestimated during this period considering the existence of silent, paucisymptomatic, or atypical forms of the disease. Serological tests mayallow the detection of subjects with mild symptoms or even without any symptoms, or to retrospectively diagnose unconfirmed symptomatic cases. To assess the burden of COVID-19 in PLWHIV, we performed an epidemiological survey after the first lifting of stringent containment measures in our HIV reference center in Northern France. At the first visit of their routine follow-up, PLWHIV completed a questionnaire about risk exposures and symptoms consistent with COVID-19, and benefited from a SARS-CoV-2 serological test. 

## 2. Materials and Methods

### 2.1. Patients and Hospital Setting

We conducted a single center cohort study in Tourcoing Hospital, over 4 months (1 April 2020 through 1 August 2020). Tourcoing Hospital is a reference center for care of PLWHIV. A cohort of 2069 PLWHIV is currently followed in our center. All HIV-infected patients who benefited from a follow-up visit during the study period were asked to answer the questionnaire and performa COVID-19 serological test.

### 2.2. Data Collection

Demographic data and characteristics of all included patients were collected using clinical database (Nadis) [12]. The following data on HIV infection were recorded: the year of HIV diagnosis, the nadir of CD4 cell count, the zenith of HIV viral load, HIV disease staging according to CDC, the duration of undetectability, CD4 cell count, and plasma HIV viral load at the time of inclusion. History of antiretroviral treatment was collected: number of successive antiretroviral regimens, duration of antiretroviral therapy, and current antiretroviral treatment. Other underlying clinical conditions were also registered. The following data were recorded from the questionnaire: history of travel since 1 January, contact with a person infected with COVID-19, and symptoms or clinical signs consistent with COVID-19. Results of COVID-19 PCR assay previously performed were recorded. 

### 2.3. Biological Assays

All included patients had one COVID-19 serological assay performed in the Tourcoing Hospital laboratory. The method implemented for the detection of anti-SARS-CoV-2 antibodies was the serological assay Roche^®^ (Basel, Switzerland) Elecsys Anti-SARS-CoV-2. This assay detects total antibodies (both IgM and IgG) to SARS-CoV-2 [13]. Before analyses on patients’ sera, calibration was performed and quality controls were passed as per manufacturers’ instructions. Signal cutoff index (COI) of ≥1.0 was interpreted as reactive and a COI of <1.0 was interpreted as non-reactive in accordance with manufacturers’ product insert.

### 2.4. Definitions and Endpoints

A confirmed case of COVID-19 was defined as a patient with at least one positive result of PCR assay or serological assay. For patients with multiple samples, only the first positive one was retained. Patients with only negative samples were considered uninfected. The primary goal was to assess the COVID-19 prevalence in the study population defined by the number of confirmed cases (by PCR or serological assay) divided by the total number of patients included. The secondary goal was to investigate the association between COVID-19 confirmed cases and demographics (age and sex), occupational and private contact with COVID-19 infected patients, HIV infection characteristics, and prior symptoms consistent with COVID-19 infection.

### 2.5. Statistical Analysis

Continuous variables were expressed as mean and standard deviation. They were compared using the Mann–Whitney test. Categorical variables were expressed as numbers and percentages. They were compared using the Fisher’s exact test. Differences between groups were considered to be significant for variables yielding a *p* value < 0.05. The incidence rate of COVID-19 was assessed, with 15 February defined as the beginning of the epidemic, and contamination periodconsidered as being up to 15 days before serology, as contamination was supposed to be uniform over this period. An incidence curve of symptomatic cases was also performed. All statistical analysis were performed using R software, version 4.0.5.

## 3. Results

### 3.1. Demographic and Clinical Data

**Figure 1:**
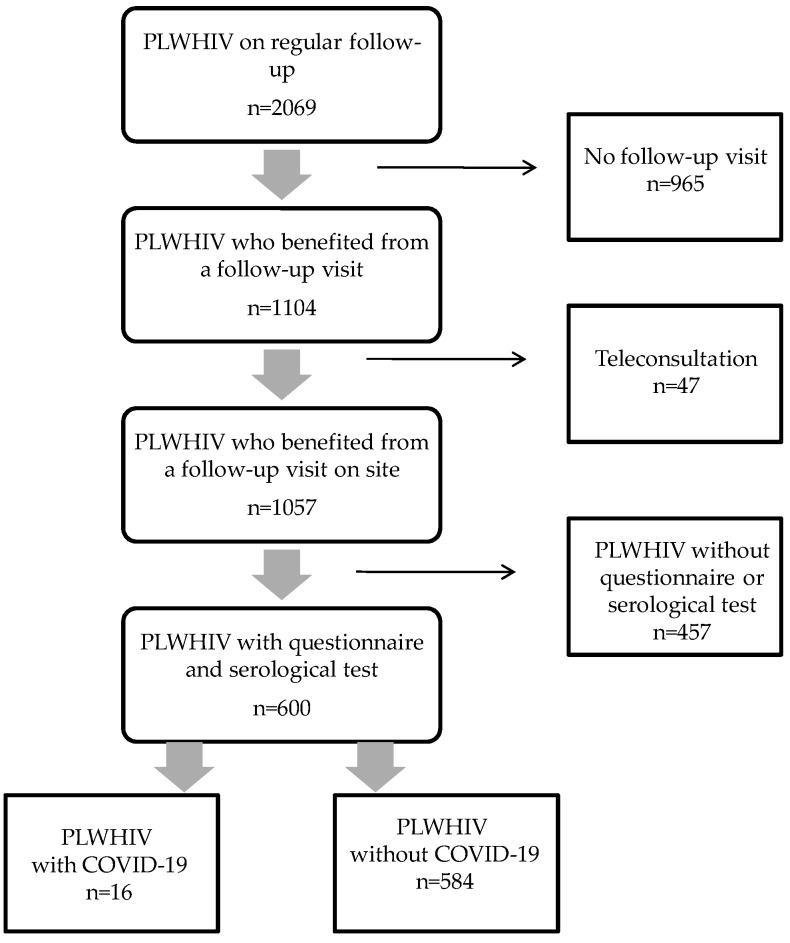
Patient flowchart.

In Figure 1, the flow chart of the study inclusion process is reported. During the study period, 1057 patients benefited from at least one follow-up visit at our center. A total of 600 HIV-1-infected patients were included. The study population did not differ significantly from the overall population on regular follow-up in our clinic, particularly regarding gender (male proportion: 74% vs. 73.4%, *p* = 0.7), age (48.8 ± 12.6 vs. 49.4 ± 12.7 years, *p* = 0.27), nadir of CD4 cell count (265 ± 183 vs. 256 ± 176 cells/mm^3^, *p* = 0.46), zenith of HIV viral load (485,132 ± 1,352,233 vs. 491,522 ± 1,469,183 copies/mL, *p* = 0.50). Among the overall population of PLWHIV on regular follow-up, the rate of hospitalization of confirmed COVID-19 cases was 5%, with 2% of patients admitted to the ICU. In our cohort of PLWHIV, the case fatality rate of COVID-19 was 1.2%.

Demographic and clinical characteristics of our patients, depending on COVID-19 status, are shown in Table 1. Our patients were predominantly male (74%) with a mean age of 49 ± 13 years. The mean duration of HIV infection was 12.1 ± 8.1 years. One hundred and sixteen patients (19%) had a history of AIDS-defining condition (CDC stage C). All our patients were currently treated with combination antiretroviral therapy, leading to an undetectable HIV viral load (<20 copies/mL) in 534 patients (89%). The mean duration under antiretroviral therapy was 9.7 ± 5.9 years. Antiretroviral regimens included nucleoside reverse transcriptase inhibitors (*n* = 526, 88%), non-nucleoside reverse transcriptase inhibitors (*n* = 185, 31%), integrase strand transfert inhibitors (*n* = 395, 66%), protease inhibitors (*n* = 87, 15%), and maraviroc (*n* = 11, 2%). Current CD4 lymphocytes count was above 200 cells/mm^3^ in 597 subjects (99%). The main associated comorbidities were a cardiovascular disease in 162 patients (27%), and diabetes mellitus in 44 patients (7%). Obesity was observed in 104 patients (17%). Seventeen patients reported another factor of immunodepression (3%). As shown in Table 1, there was no significant difference between SARS-CoV-2 positive and negative patients for age, gender, type and number of co-morbidities, time with HIV infection, the nadir of CD4-cell counts, current CD4 counts and viral load, duration, and type of anti-HIV therapy.

### 3.2. Exposure Factors, Symptoms, and Diagnostic Tests

SARS-CoV-2 exposure factors and symptoms depending on COVID-19 status are summarized in Table 2. Concerning exposure factors to COVID-19, 82 patients (14%) reported a close contact with a confirmed COVID-19 case, 33 (5%) reported a travel history abroad since 1 January 2020, and 24 patients (4%) were healthcare workers. Close contact with a confirmed COVID-19 case was the only exposure factor associated with COVID-19 diagnosis (40% inCOVID-19 confirmed cases vs. 13% in non COVID-19 cases, *p* = 0.01).

The most common symptoms consistent with COVID-19 in the study population were myalgia (19%), fatigue (18%), cough (17%), fever (13%), and headaches (12%). The time from symptoms onset to the first HIV follow-up visit was longer than 8 weeks in 65% of our patients. At least one symptom consistent with COVID was reported by 67% of the patients with confirmed COVID-19 and 32% of those without COVID-19 (*p* = 0.021). Among patients with confirmed COVID-19, five (31%) were asymptomatic. One patient was hospitalized because of SARS-CoV-2 pneumonia, with no need for intensive care admission.

Thirteen patients (2%) had a COVID-19 PCR assay performed on a nasopharyngeal swab before their consultation. The test was positive in three cases. Sixteen patients tested positive for SARS-CoV-2 total antibodies. All three patients who had a history of positive RT-PCR result, had a positive serological test.

### 3.3. Prevalence and Incidence of COVID-19

**Figure 2:**
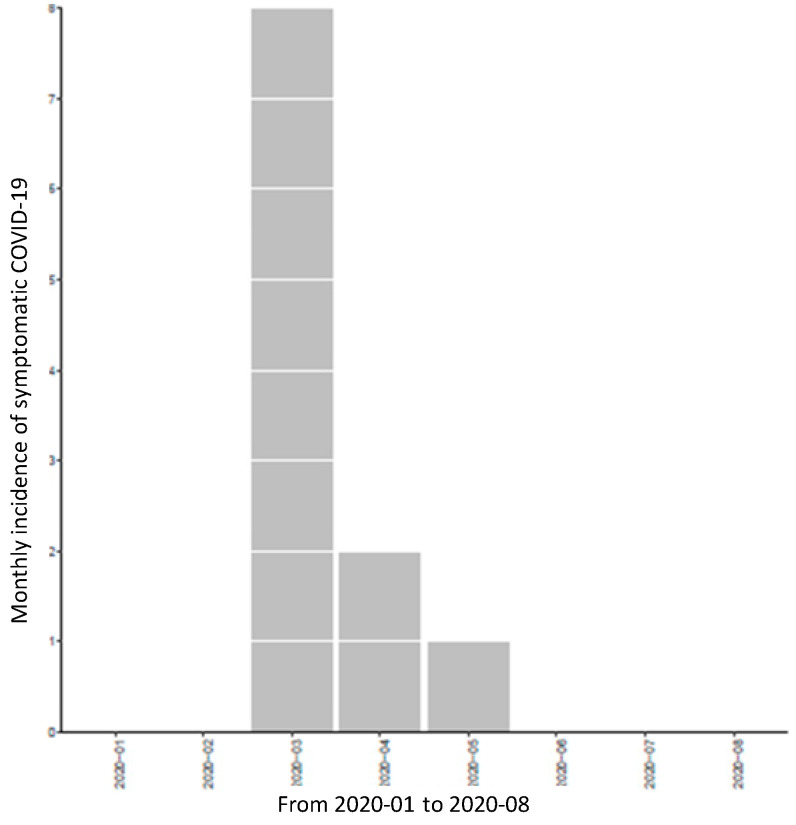
Epidemic curve of symptomatic COVID-19.

Among our 600 patients included, 16 had a confirmed diagnosis of COVID-19. The overall SARS-CoV-2 seroprevalence was 2.7%. Incidence was estimated at 0.16 per 100 patients·week from the beginning of the epidemic in France to the end of the survey. Figure 2 shows the epidemic curve of symptomatic COVID-19. 

## 4. Discussion

In our population of HIV-1-infected patients seen in routine clinical practice, the COVID-19 prevalence observed after the lifting of stringent containment measures was 2.7%. Performance of SARS-CoV-2 serological tests allowed the retrospective diagnosis of COVID-19 in eight symptomatic patients who did not benefit from RT-PCR assay at the time of symptoms onset and in five asymptomatic patients. All three patients with a previously confirmed diagnosis of COVID-19 by positive SARS-CoV-2 PCR assay, had also a positive serological test.

To date, the published data analyzing the SARS-CoV-2 prevalence in the French general population are scarce. Grzelak L et al. examined SARS-CoV-2 seroprevalence in serum samples collected from healthy blood donors in the North of France from20 March to 24 March 2020 [13,14]. They found a positivity rate of 1 to 3%, similar to the positivity rate in our cohort of PLWHIV. However, their study concerned only asymptomatic subjects. Indeed, eligibility criteria for blood donation included the absence of recent signs of infection. Furthermore, the study was conducted at a time when SARS-CoV-2 had not yet circulated to a large extent. 

Salje H. et al. estimated the burden of SARS-CoV-2 in France at the time of lifting stringent containment measures, using a suite of modeling analyses [15]. They concluded that 5.3% of the French population (range: 3.3 to 9.3%) will have been infected after the early 2020 pandemic. They estimated the proportion of infected patients in the Northern area of France at 5.7% (range: 3.5 to 10.3%). Taking these results into account, the prevalence of COVID-19 in our cohort of PLWHIV appeared to be half that of the overall population after the first wave of the COVID-19 pandemic in France. 

Data concerning the epidemiology of SARS-CoV-2 infection in PLWHIV are limited. Charre C et al. in a study conducted in the South of France found a similar COVID-19 attack rate in PLWHIV as in the general population during the early 2020 pandemic [7]. In their study, confirmed cases concerned almost exclusively symptomatic cases and were limited in the HIV subgroup. In our cohort, the majority of cases were diagnosed with serological assays. Assessment of antibody responses to SARS-CoV-2 in patients with COVID-19 revealed seroconversion in 95% to 100% of patients within 3 weeks after symptoms onset [16,17]. However, the proportion of positive samples for SARS-CoV-2 antibodies in COVID-19 infected patients varied depending on the sampling times from symptoms onset [14]. The evolution of SARS-CoV-2 antibodies 35 days after symptoms onset has not been properly evaluated [18]. Since delays from symptoms onset to follow-up visit often exceed 60 days in our cohort, it could have led to an underestimation of COVID-19 frequency among our patients. Moreover, antibody responses may be lower in PLWHIV. Huang et al. described virological and serological features of COVID-19 cases in people living with HIV in Wuhan City [19]. The proportion of recovered COVID-19 patients who tested positive for SARS-CoV-2 antibodies reached 79%. The level of viral load seemed to influence the antibody response against SARS-CoV-2. 

In our cohort of PLWHIV, the estimated incidence of COVID-19 was 0.16 per 100 patients·week. This result is difficult to compare to the incidence in the general population. During the first wave of the COVID-19 pandemic, the testing national strategy in France was centered on symptomatic infections, especially hospitalized patients, leading to an underestimation of diagnosis during this period. In France, the Sentinelles network monitors cases of influenza-like illnesses and acute respiratory infections in general practice [20]. At the beginning of the epidemic, a correlation was found between the excess cases reported by this network and the confirmed cases of COVID-19 in the various regions of France [21]. In early March, consultations in excess already reached 175 per 100,000 in Northern France. The surveillance of influenza-like illnesses and acute respiratory infections helped to estimate the impact of the COVID-19.

In our study population of PLWHIV, none of the comorbidities, including obesity, diabetes, and cardiovascular diseases were associated with COVID-19. More than a risk of acquisition of COVID-19, these comorbidities, especially cardiovascular disease related to atherosclerosis have been identified as poor prognosis factors in COVID-19 patients [22].

In our population of PLWHIV, none of the variables linked to HIV infection, including the time of HIV infection, CDC stage, the nadir of CD4 cell counts, current CD4 count, and viral load were associated with COVID-19. Current antiretrovirals did not differ between SARS-CoV-2 positive and negative patients. Our study was underpowered to draw strong conclusions. However, a previous prospective cohort study conducted in Italy led to similar findings [23]. A study conducted in Wuhan identified being over the age of 50 and discontinuation of antiretroviral therapy as risk factors for COVID-19 in PLWHIV [19]. Lopinavir/ritonavir and tenofovir showed in vitro antiviral activity on SARS-CoV-2. However, taking available evidence into account, no antiretroviral drug demonstrated a preventing role against COVID-19 [24].

Close contact with a confirmed COVID-19 case was the only factor associated with COVID-19 acquisition in our cohort of PLWHIV. As in the general population, risk exposition during occupational and recreational activities, adherence to lock-down and social-distancing procedures are determinant for the risk of COVID-19 acquisition. In France, all HIV-infected patients were initially identified at risk of COVID-19. After the publication of several case series of HIV-patients with COVID-19 from China, Spain, Germany, Italy, and the United States, no evidence for a different disease course in people with HIV than in HIV-negative people was found [25,26,27,28,29,30]. Only HIV-patients with low CD4T-cell count (<200/mm^3^), or those not receiving antiretroviral treatment are still considered at risk for a more severe disease presentation, following the European guidelines [31]. Although the majority of our patients were on effective antiretroviral treatment with undetectable viral load, and very few had a CD4 T-cell count bellow 200/mm^3^, many of them were older than 50 years of age and suffered from at least one comorbidity. The lower COVID-19 prevalence observed in our cohort of PLWHIV than in the general population could be explained by PLWHIV being more cautious about social distancing and showing better awareness of containment measures.

Symptoms consistent with COVID-19 were frequent both in SARS-CoV-2 positive and negative PLWHIV. A similar observation was made in the general population [32]. The most frequent symptoms in PLWHIV with confirmed COVID-19 were fatigue, myalgia, headache, fever, cough, like previously described in non-HIV-infected subjects. The rate of hospital admission of our COVID-19 confirmed cases was 6%. None of the patients were admitted to the ICU. Finally, in our population of PLWHIV, infection was asymptomatic in a large proportion of subjects. Similarly, Maggiolo F. et al. reported 18% of completely asymptomatic patients in a single-center Italian PLWHIV cohort [23]. The high proportion of asymptomatic cases in PLWHIV should be taken into account in future epidemiological studies.

Our study has several limitations. First, patients who died from COVID-19 were not analyzed. Second, by including outpatients, most critically ill patients, especially those requiring a long hospital stay for COVID-19 may also have been excluded. However, the prolonged period of inclusion limited this selection bias. Third, not all the patients who benefited from a follow-up visit during the study period, completed the survey, and had a serological test performed, leading to potential selection bias. The relatively high proportion of patients included could reduce this risk. Finally, the majority of our patients had an undetectable viral load, and high CD4 count, preventing any solid conclusion about epidemiologic data in the population who had uncontrolled HIV infection.

## 5. Conclusions

In conclusion, the prevalence of COVID-19 in PLWHIV was two time slower than in the general population. Only close contact with COVID-19 infected subjects was associated with acquisition of SARS-CoV-2. None of the variables linked to HIV infection were determinant for the risk of COVID-19 infection.

## Figures and Tables

**Table 1:** Demographic and clinical characteristics of our patients, depending on the COVID-19 status.

Patients Characteristics	Total *n* = 600	COVID-19 Negative Patients *n* = 584	COVID-19 Confirmed Cases *n* = 16	*p*-Value
Age (years)	49 ± 13	49 ± 13	49 ± 15	0.99
Male gender (%)	446 (74.3)	432 (73.8)	14(93.3)	0.13
Obesity (BMI ≥ 30 Kg/m^2^)	104 (17.3)	101 (17.3)	3 (20)	0.73
Pregnancy	1 (0.17)	1 (0.17)	0 (0)	1.00
Comorbidities				
Other factor of immunodepression	16 (2.7)	15 (2.6)	1 (6.7)	0.34
Diabetes melitus	44 (7.3)	43 (7.4)	1 (6.7)	1.00
Cardiovascular diseases	162 (27.0)	158 (27.0)	4 (26.7)	1.00
Chronic hepatitis	66 (11.0)	64 (10.9)	2 (13.3)	0.62
Chronic pulmonary disease	62 (10.3)	62 (10.6)	0 (0.0)	0.39
Chronic kidney disease	32 (5.3)	32 (5.5)	0 (0.0)	1.00
HIV infection				
CDC stage				0.07
A	372 (62)	361 (61.7)	11 (73.3)	
B	109 (18.2)	108 (18.5)	1 (6.7)	
C	116 (19.3)	114 (19.5)	2 (13.3)	
Nadir of CD4 cell count (/mm^3^)	265 ± 183	266 ± 183	243 ± 156	0.60
CD4 cell count < 200/mm^3^	10 (1.7)	9 (1.5)	1 (7.1)	0.39
Current CD4 cell count	707 ± 300	709 ± 302	630 ± 220	0.21
Current viral load	242 ± 2978	232 ± 2998	657 ± 1969	0.46
Viral load < 20 copies/mL	532 (89)	521 (89)	11 (79)	0.20
Duration of HIV (years)	12.1 ± 8.1	12.1 ± 8.1	12.6 ± 10.3	0.84
Current antiretroviral therapy				
TDF/TAF	365 (61)	354 (61)	11 (73)	0.46
Lopinavir	5 (0.8)	5 (0.9)	0 (0.0)	1.00

Mean (standard deviation) and number (%).

**Table 2:** SARS-CoV-2 exposure factors and symptoms depending on the COVID-19 status.

Characteristics	Total *n* = 600	COVID-19 Negative Patients *n* = 584	COVID-19 Confirmed Cases *n* = 16	*p*-Value
Exposure factors:				
Travel abroad since January 2020	66 (11)	63 (10.8)	3 (20.0)	0.22
Contact with a COVID-19 case	82 (13.7)	76 (13.0)	6 (40.0)	0.01
Healthcare worker	24 (4)	22(3.8)	2 (13.3)	0.12
Symptoms:	194 (32.3)	184 (31.5)	10 (66.7)	0.02
Fever	76 (12.7)	70 (12.0)	6 (40)	0.007
Cough	105 (17.5)	98 (16.8)	7 (46.7)	0.008
Myalgia	112 (18.7)	103(17.6)	9 (60)	<0.001
Fatigue	110 (18.3)	101 (17.3)	9 (60)	<0.001
Headache/dizziness	70 (11.7)	64 (10.9)	6 (40)	0.004
Digestive disorders	49 (8.2)	48 (8.2)	1 (6.7)	1.00
Dyspnea	47 (7.8)	43 (7.3)	4 (26.7)	0.02
Anosmia	15 (2.5)	11 (1.9)	4 (26.7)	<0.001
Loss of taste	17 (2.8)	14 (2.4)	3 (20.0)	0.007
SARS-CoV-2 PCR test:	13 (2.2)	10 (1.7)	3 (20.0)	0.03
Positive	3 (0.5)	0 (0.0)	3 (20.0)	<0.001

All numbers represent the number of patients (percent).

## Data Availability

Data are available under request by email to corresponding author.
